# Exercise‐Induced growth hormone during acute sleep deprivation

**DOI:** 10.14814/phy2.12166

**Published:** 2014-10-03

**Authors:** Kevin Ritsche, Bradly C. Nindl, Laurie Wideman

**Affiliations:** 1 Department of Kinesiology, University of North Carolina‐Greensboro, Greensboro, North Carolina, USA; 2 Department of Exercise Physiology, Winston‐Salem State University, Winston‐Salem, North Carolina, USA; 3 US Army Institute of Public Health, US Army Public Health Command, Aberdeen, Proving Ground, Aberdeen, Maryland, USA

**Keywords:** anaerobic, GH, High‐intensity exercise, sleep loss, sprint exercise

## Abstract

The effect of acute (24‐h) sleep deprivation on exercise‐induced growth hormone (GH) and insulin‐like growth factor‐1 (IGF‐1) was examined. Ten men (20.6 ± 1.4 years) completed two randomized 24‐h sessions including a brief, high‐intensity exercise bout following either a night of sleep (SLEEP) or (24‐h) sleep deprivation (SLD). Anaerobic performance (mean power [MP], peak power [PP], minimum power [MinP], time to peak power [TTPP], fatigue index, [FI]) and total work per sprint [TWPS]) was determined from four maximal 30‐sec Wingate sprints on a cycle ergometer. Self‐reported sleep 7 days prior to each session was similar between SLEEP and SLD sessions (7.92 ± 0.33 vs. 7.98 ± 0.39 h, *P *=**0.656, respectively) and during the actual SLEEP session in the lab, the total amount of sleep was similar to the 7 days leading up to the lab session (7.72 ± 0.14 h vs. 7.92 ± 0.33 h, respectively) (*P *=**0.166). No differences existed in MP, PP, MinP, TTPP, FI, TWPS, resting GH concentrations, time to reach exercise‐induced peak GH concentration (TTP), or free IGF‐1 between sessions. GH area under the curve (AUC) (825.0 ± 199.8 vs. 2212.9 ± 441.9 *μ*g/L*min, *P *<**0.01), exercise‐induced peak GH concentration (17.8 ± 3.7 vs. 39.6 ± 7.1 *μ*g/L, *P *<**0.01) and ΔGH (peak GH – resting GH) (17.2 ± 3.7 vs. 38.2 ± 7.3 *μ*g/L, *P *<**0.01) were significantly lower during the SLEEP versus SLD session. Our results indicate that the exercise‐induced GH response was significantly augmented in sleep‐deprived individuals.

## Abstract (precis)

Human growth hormone release is heavily influenced by sleep and exercise. Our study shows that sleep deprivation dramatically augments the exercise‐induced human growth hormone response.

## Introduction

Human growth hormone (GH) is secreted from the anterior pituitary gland, which is heavily regulated by growth hormone‐releasing hormone (GHRH) and somatostatin (SMS). In addition, GH output is mediated by ghrelin (GHS) and insulin‐like growth factor‐1 (IGF‐1). GH is secreted in a pulsatile fashion, with the strongest physiologic stimuli being sleep and exercise.

Humans spend approximately one‐third of their lives sleeping (Morris et al. 2012). The major secretory GH pulse occurs just after sleep onset and continues to rise during the first 4 h. Most GH release occurs during non‐rapid‐eye movement (NREM) sleep within the slow‐wave sleep (SWS) phase with little GH secreted during rapid‐eye movement (REM) sleep (Takahashi et al. 1968; Parker et al. 1969; Sassin et al. 1969; Holl et al. 1991; Van Cauter et al. 1992a,b). Individuals undergoing some form of sleep deprivation, such as doctors, nurses, shift workers and military personnel, offer many of our important societal services. Additionally, athletes and coaches believe that sleep is essential for peak physical performance. There are many situations where sleep is disturbed prior to an athletic event including travel, changes in time zones and anxiety. Exponentially, student‐athletes in higher education are forced to accommodate their study schedule for athletic events, resulting in altered sleep habits and/or sleep loss in order to study for exams. In these situations, the question arises as to how exercise may be used to neutralize the physiological effects of sleep deprivation in regard to GH.

Sleep deprivation can alter hypothalamus and pituitary function, which de‐synchronizes GH release timing (Van Cauter et al. 1992a; Wiebel et al. 1997; Brun et al. 1998; Brandenberger and Wiebel 2004; Everson and Crowley 2004). During complete sleep deprivation of 24–36 h, GH release is attenuated and in some cases, absent (Takahashi et al. 1968; Sassin et al. 1969; Karacan et al. 1971; Brun et al. 1998; Brandenberger and Wiebel 2004). Brun et al. (1998) reported that GH release was dramatically reduced during a 36‐h sleep‐deprived session with the most noticeable decrease in nocturnal GH peak values (28.2 ± 17.9 µg/L vs. 5.5 ± 3.4 µg/L, for control and sleep deprivation sessions, respectively). Peak nocturnal GH secretory bursts were observed in the subjects between 23:00 and 2:30 h during the control session. During the sleep‐deprived session, the nocturnal peak amplitude and total GH area under the curve (AUC) were dramatically reduced although the total number of GH peaks during the 24‐h sampling period was similar between the control and sleep‐deprived session. Futhermore, Brandenberger et al. (2000) reported that 24‐h GH pulsatility profiles were different between habitual sleepers and adapted night shift workers. Not only was nocturnal GH release lower in adapted night shift workers, but 24‐h GH pulsatility was more frequent, sporadic and unpredictable throughout waking hours. Based on these observations, we assume that complete sleep deprivation can attenuate the GH response the morning after and this is likely the result of of the disturbance of the sleep‐wake cycle.

Exercise is a proven stimulus of GH release and an acute bout of exercise stimulates a significant GH pulse (Sutton and Lazarus 1976; Bunt et al. 1986; Felsing et al. 1992; Weltman et al. 1992, 2006; Chwalbinska‐Moneta et al. 1996; Pritzlaff et al. 1999, 2000; Wideman et al. 1999, 2000a,b, 2002; Pritzlaff‐Roy et al. 2002; Godfrey et al. 2003). Although there is large interindividual variation in exercise‐induced GH release, it is a reproducible measure within subjects (Stokes et al. 2003; Makimura et al. 2008). Multiple studies have shown that shorter bouts of high‐intensity exercise can elicit an elevated growth hormone response (Nevill et al. 1996; Kanaley et al. 1997; Stokes et al. 2002a,b, 2004, 2005). Peak GH release occurs within ~30–45 min after the initiation of sprint exercise and a single 6‐sec sprint can augment GH release (Stokes et al. 2002a), although a slightly longer 30‐sec sprint enhances GH release further (Nevill et al. 1996; Stokes et al. 2002b, 2005).

To our knowledge, no one has examined the effects of acute (24‐h) sleep deprivation on exercise‐induced GH release using short‐term, high‐intensity exercise. The two studies that have examined the effects of exercise‐induced GH release during complete (24‐h) sleep deprivation and exercise‐induced GH release during partial sleep deprivation have yielded inconsistent results largely due to methodological differences involving protocols that only partially disturbed the sleep‐wake cycle (Mougin et al. 2001; Abedelmalek et al. 2013). Considering GH release begins around the onset of sleep, partial sleep deprivation may still provide sufficient rest to elicit a normal nocturnal GH release and thus, limit the alterations observed in subsequent exercise‐induced GH release. Therefore, the purpose of this study was to investigate the effects of acute sleep deprivation on subsequent exercise‐induced GH release. Since we anticipated that GH production and storage would continue during a period of sleep deprivation followed by a subsequent bolus of release upon stimulation, we hypothesized that GH release will be augmented in response to short‐term, high‐intensity exercise following a 24‐h period of continuous sleep deprivation.

## Methods

### Subject characteristics

Ten men (9 Caucasians and 1 African‐American), ages 18–22 years (20.6 ± 1.4 years) with a mean body mass index of 26.6 ± 2.5 kg/m^2^, and body fat percentage of 20.9 ± 6.8%, completed the study. On average, subjects reported participating in high‐intensity activity 1 day per week and spent 4–6 h per week participating in recreational physical activity and reported minimal occupational physical work. All subjects provided written informed consent in accordance with the institutional review board at the University of North Carolina at Greensboro and Winston‐Salem State University. All subjects underwent a strict preliminary screening session prior to inclusion. Subjects were excluded if they: (1) had a body fat percentage >30%, (2) reported a history of hematological, renal, hepatic, metabolic or thyroid dysfunction, (3) were currently on a caloric restriction program (diet) or taking any medications that promoted weight loss, (4) currently smoked or had quit smoking within the previous 3 months, (5) had documented sleep disturbances or irregular sleeping patterns such as night shift work or recreational habits, (6) had completed transmeridian travel within the last month, or (7) participated in greater than 10 hours of recreational activities (swimming, basketball, jogging, cycling etc.) per week or were involved in any type of sprint training within the last 6 months. Self‐reported sleep logs were kept for seven days prior to both 24‐h laboratory sessions. Epworth Sleepiness scales were used to screen for baseline excessive subjective sleepiness and/or irregular sleeping habits (Johns 1991).

Individuals who met the inclusion criteria completed a brief familiarization session on an electronically braked cycle ergometer (Lode Excalibur Sport, Lode BV, Gronignen, The Netherlands). Equipment used to collect metabolic measures using standard open circuit spirometry (TrueOne^®^ metabolic measurement system from ParvoMedics [(Sandy, UT)] during exercise was fitted during this familiarization session, but no gases were collected.

### Experimental design

**Figure 1.**
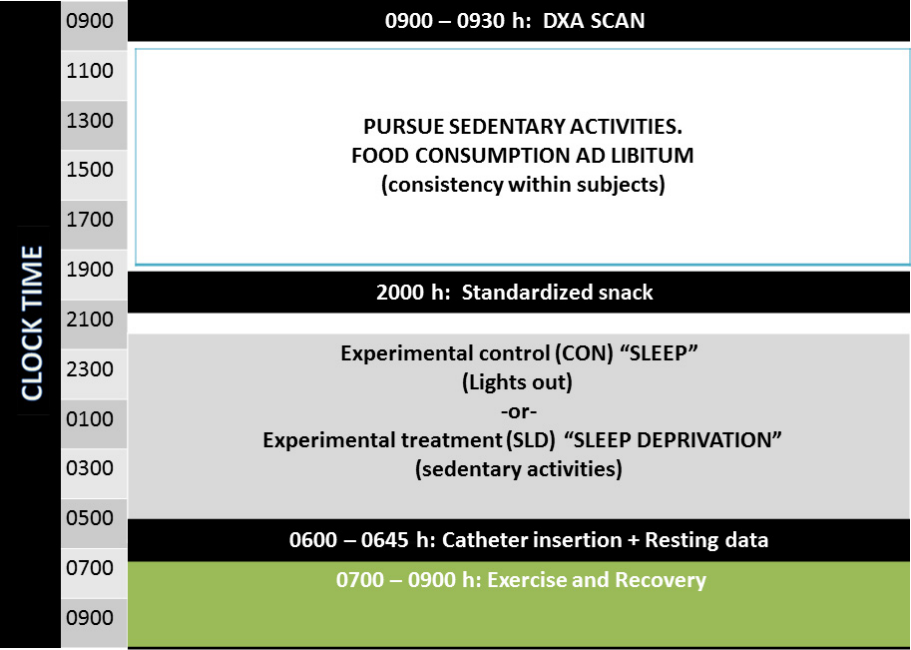
Experimental protocol showing the 24‐h laboratory sessions. The design was counter‐balanced and randomized between sessions.

Subjects completed one baseline 24‐h laboratory session where their acute exercise response was measured following a night of sufficient rest and compared to the identical exercise‐induced response following a 24‐h sleep deprivation session (Fig. 1). Subjects were instructed to refrain from exercise 48 h prior to their randomized laboratory session. In order to control for order effects, a counterbalanced design was used in which half the subjects performed the sleep deprivation session (SLD) first and the other half performed the control sleep session (SLEEP) first. Testing days of the week were standardized within subjects and separated by 3 weeks. For both sessions, subjects reported to the Exercise Physiology laboratory at 0800 h. Ambient light and temperature in the laboratory were held constant between 0800 and 2200 h. During the SLEEP session, subjects were allowed adequate rest in a light‐controlled environment (2200–0600 h) and investigators only entered the room briefly (<5 min) to check on the subject. If a subject was unable to remain asleep the entire period, they were asked to remain in bed in darkness until an investigator beckoned them at 0600 h. During the SLD session, subjects were sleep‐deprived by only allowing them to rest in wakeful state or pursue intellectual activities between the hours of 2200 to 0600 h. They were kept sedentary throughout the session, but were allowed to read, study and watch movies, and researchers maintained social interaction with the subjects throughout the entire sleep‐deprived session. During this time, the ambient light in the laboratory was unchanged from daylight hours.

### Body composition measurements

During the first randomized laboratory session, subjects had their body composition assessed via whole body dual‐energy X‐ray absorptiometry (DXA) scan (Lunar‐Prodigy Advance Plus, GE) at 0900 h. All scans were performed in fan beam mode using the thick scan mode (recommended by GE for research purposes; scan time was approximately 20 min). Fat mass (FM) and fat‐free mass (FFM) were measured for the whole body and regional areas.

### Standardized caloric intake

A detailed dietary log was recorded for each subject 72 h prior to and through the conclusion of each session and then analyzed for total calories using myfitnesspal.com^®^. No caffeine or alcohol was allowed for the 48 h preceding laboratory studies through the completion of the 24‐h session. Daily caloric intake was standardized during each session for all subjects based on their estimated basal metabolic rate using the Harris Benedict Principle (Harris and Benedict 1918) (BMR = [66.4730 + 13.7516 × weight {kg}] + [5.0033 × height {cm}]−[6.7550 × age {years}] * [physical activity]). Each subject was instructed to eat within ~600 kcal of their predetermined daily caloric intake each time they came to the laboratory and to stay consistent with their meals during each subsequent session. At 2000 h, subjects were given a standardized ~600 kcal snack that had a macronutrient content of ~45% carbohydrate, ~20% protein and ~35% fat. Following their snack, subjects fasted throughout the remainder of their session (2000–0900 h).

### Exercise testing protocol

Upon catheter insertion at 0600 h, subjects rested passively in a chair while flow meters and gas analyzers were calibrated for collection of metabolic measurements using open circuit spirometery [(TrueOne^®^ metabolic measurement system from ParvoMedics (Sandy, UT). After 20 min of passive rest, subjects were fitted with their headgear and mouth pieces to breath through to initiate gas collection while they continued to rest for another 25 min to insure accurate resting metabolic rate (RMR) data collection postcatheter insertion. Forty‐five minutes into their passive rest period subjects, completed a 5‐min standardized submaximal warm‐up that consisted of pedaling on a cycle ergometer at: 60 watts (W) for 4 min, 80 W for 30 sec and 100 W for 30 sec. At 0700 h, subjects completed four maximal 30‐sec sprints on a cycle ergometer against an electronically applied resistance equivalent to 7.5% of their body weight (kg). Each sprint was followed by 4 min of active recovery on the cycle ergometer at 50 W.

### Blood sampling and analysis

**Figure 2.**
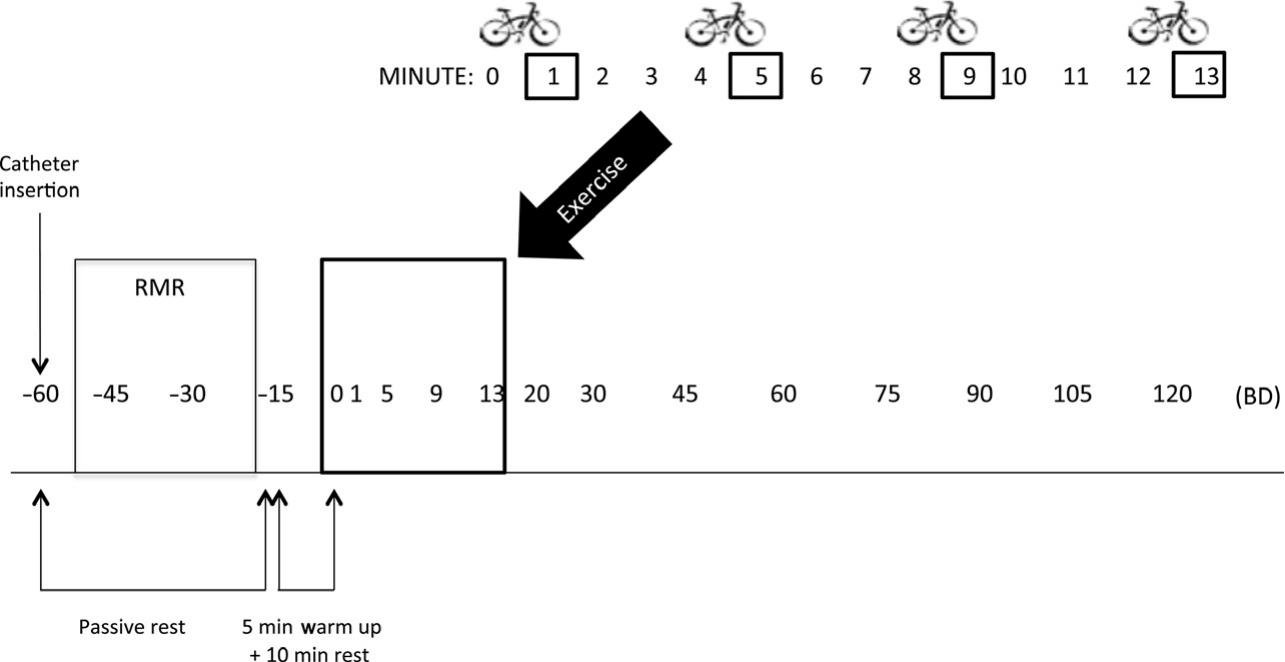
Blood draw profile. Samples were collected over 3‐h that included ~30 min of resting metabolic rate (RMR) data collection and blood draws that occur every 15 min on average, with more frequent sampling around the 13‐min exercise session. BD, blood draw. There were 17 blood draws in total.

Blood was collected by a trained technician through a catheter inserted into an arm vein in the antecubital space. Catheter patency was maintained by displacing the blood in the catheter with isotonic saline at regular intervals. A heating pad was placed over the antecubital area in order to minimize peripheral venoconstriction and maximize patency during the postexercise period. Blood samples were taken at seventeen different time points (Fig. 2) and, on average, every 15 min (~Q15) for 180 min, with more frequent sampling just prior to initiating the first exercise sprint and immediately after the 4^th^ exercise sprint a postexercise blood sample was collected. Blood samples were allowed to clot at room temperature for 30 min. Samples were then centrifuged at 3000 rpm for 15 min at 4°C. Serum was extracted and pipetted into microcentrifuge tubes and stored at −80°C until subsequently analyzed.

Growth hormone from all time points were determined in duplicate using a human GH enzyme‐linked immunosorbant assay (MP Biomedicals, Solon, OH). The minimum detectable dose of this assay was 0.5 *μ*g/L and the intra‐assay variance was 2.2–2.9%. Insulin‐like growth factor‐1 (IGF‐1) immediate preexercise and 120 min postexercise onset (~106 min postexercise) was assayed in duplicate using an in vitro enzyme‐linked immunosorbent assay (Raybiotech, Norcross, GA). The minimum detectable dose of this assay was 0.1 *μ*g/L and the intra‐assay variance was <10%. To eliminate interassay variance, all samples from a single subject were assayed within the same plate.

### Statistical analysis

Sleep (self‐reported average amount of sleep per night) patterns prior to and during each session were examined using a paired‐sample t‐test. Fifteen second breath‐by‐breath averages of oxygen consumption (VO_2_), carbon dioxide (VCO_2_), metabolic equivalents (METS), respiratory rate (RR), ventilation (VE), tidal volume (V_T_) and respiratory exchange ratio (RER) were calculated from open circuit spirometery for 30 min prior to exercise through the end of exercise (Q‐30 to Q15; 0630 h–0715 h). A paired‐sample t‐test was used to determine whether there were any differences in physiological data during rest and exercise values between the SLD and SLEEP sessions. When data was non‐normally distributed, a nonparametric Wilcoxin test for two related samples was used to adjust for the skewedness.

Analysis of anaerobic performance data was done in a stepwise fashion. First, mean power (MP), peak power (PP), minimum power (MinP), time to peak power (TTPP), fatigue index (FI), and total work completed (TW) were calculated from each of the sprint exercise tests. Data descriptives were then examined for violations of test assumptions (skewedness) and those variables that were non‐normally distributed were examined using the nonparametric Wilcoxin test for 2 related samples in a preliminary analysis of each sprint between sessions (sprint 1 SLD vs. sprint 1 SLEEP). Then, a two‐way analysis of variance with repeated measures was used to determine if differences in performance existed between the SLD and SLEEP sessions (main effect – session) and between the average sprint performance score in each session (main effect – sprint). When the assumption of sphericity was violated, the Greenhouse‐Geisser correction was used.

Mean GH area under the curve (AUC) was calculated using the trapezoidal integration method. Data from each time point was included in the calculation. The AUC was calculated as previously described in detail (Stokes et al. 2003). Briefly, the intersection where the GH concentrations and time points created an ordinate was joined to form a straight line that created a trapezium. The area of each trapezium was calculated using the equation: area = (*y*^1^ + *y*^2^) × 0.5 × *d*, where *y*^1 ^+ *y*^2^ are the GH concentrations at two successive time points and *d* is the time interval between the two samples. Peak values refer to the mean of the highest measured concentrations for each individual. Resting GH was determined by the mean of the initial three time points (−60, −45, −30) preceding the exercise warm‐up. A repeated measures ANOVA, with Greenhouse‐Geisser correction, was conducted to assess whether there were differences between the average exercise‐induced GH concentration between the SLEEP and SLD sessions (main effect of session) and the response of each subject with respect to the 13 time points when blood draws occurred, starting at 0700 h just prior to exercise onset (main effect of time). Whenever mean differences were observed, mean comparisons were examined using a Wilcoxon signed‐rank test with appropriate Bonferroni corrections. Test‐retest (Pearson) correlations were used to indicate the rank order of peak GH concentration and GH AUC within and between subjects. Repeated measures ANOVA was also used to determine any differences in free IGF‐1 and lactate before and after exercise. The level of statistical significance was set at *P *<**0.05. All statistical analyses were performed using PASW for Windows, version 22.0 (Chicago, IL). All results are expressed as means ± SEM, unless otherwise noted.

## Results

### Sleep

All subjects self‐reported normal routines of work and sleep and had experienced no transmeridian travel within the last three months. Subjects did not experience sleep deprivation and demonstrated a normal sleep pattern over the previous 7 days as per their self‐reported sleep recall logs. The average amount of self‐reported sleep in the 7 days prior to each session was similar between the SLEEP and SLD sessions (7.92 ± 0.33 vs. 7.98 ± 0.39 h, *P *=**0.656, respectively). During the SLEEP session in the lab, subjects reported that they slept an average of 7.72 ± 0.14 h, which was similar to the 7 days leading up to the lab session (7.92 ± 0.33 h) (*P *=**0.166) Mean Epworth Sleepiness Scale scores were 6.4 ± 1.6, which was within the normal range (Johns 1991).

### Exercise performance

Anaerobic performance (average of all 4 sprints) did not differ between sessions (Table 1). Additionally, VCO_2_, RER, RR, exercise VO_2,_ peak VO_2_, METS, VE and VT data were all non‐normally distributed and therefore, nonparametric Wilcoxon tests for two related samples were ran to correct for the skewed data. Similar to anaerobic performance, none of the cardiorespiratory response to exercise values was significantly different between sessions (Table 2).

**Table 1.** Anaerobic performance (M ± SD).

Variable	SLEEP	SLD	*P*
Mean power (W)	528 ± 69	515 ± 75	0.175
Peak power (W)	1131 ± 198	1118 ± 183	0.681
Minimum power (W)	271 ± 69	272 ± 71	0.940
Time to peak power (sec)	1.1 ± 0.4	0.9 ± 0.2	0.135
Fatigue Index (%)	29.8 ± 5.9	29.1 ± 5.9	0.546
Total work (W)	15,850 ± 2067	15,441 ± 2264	0.175

Average sprint performance values across all sprints during the sleep (SLEEP) and sleep deprivation (SLD) sessions. *P* values were adjusted for skewedness (>1.0) of the non‐normally distributed data using the nonparametric Wilcoxin test for two related samples.

**Table 2.** Cardiorespiratory response to exercise (M ± SD).

Variable	SLEEP	SLD	*P*	Adjusted *P*†
VO_2_ (L/min)*	2.04 ± 0.43	2.04 ± 0.42	0.986	0.906
VO_2_ (mL/kg/min) *	23.4 ± 4.1	23.4 ± 3.9	0.973	0.878
METS*	6.7 ± 1.2	6.7 ± 1.1	0.936	0.959
VCO_2_ (L/min)*	2.48 ± 0.35	2.98 ± 1.14	0.181	0.415
Ventilation (VE) L/min*	90.6 ± 26.7	80.4 ± 19.3	0.209	0.139
(RER)*	1.4 ± 0.4	1.5 ± 0.5	0.724	0.374
(RR) brths/min*	33.3 ± 5.3	31.1 ± 6.9	0.260	–
Tidal Volume (VT) L/min*	2.59 ± 0.68	2.53 ± 0.58	0.672	0.443
VO_2_ peak (L/min)	3.36 ± 0.59	3.52 ± 0.66	0.388	–
VO_2_peak (mL/kg/min)	38.3 ± 4.7	39.9 ± 4.7	0.434	–
METSmax	11.0 ± 1.4	11.4 ± 1.3	0.436	–

RR, respiratory rate; RER, respiratory exchange ratio.

Exercise cardiorespiratory data by session.

* Average values over ~15 min of exercisedata collection.

† *P* values were adjusted for skewedness (>1.0) of non‐normally distributed data using the nonparametric Wilcoxin ranked signs test for two related samples.

### Growth hormone

**Figure 3.**
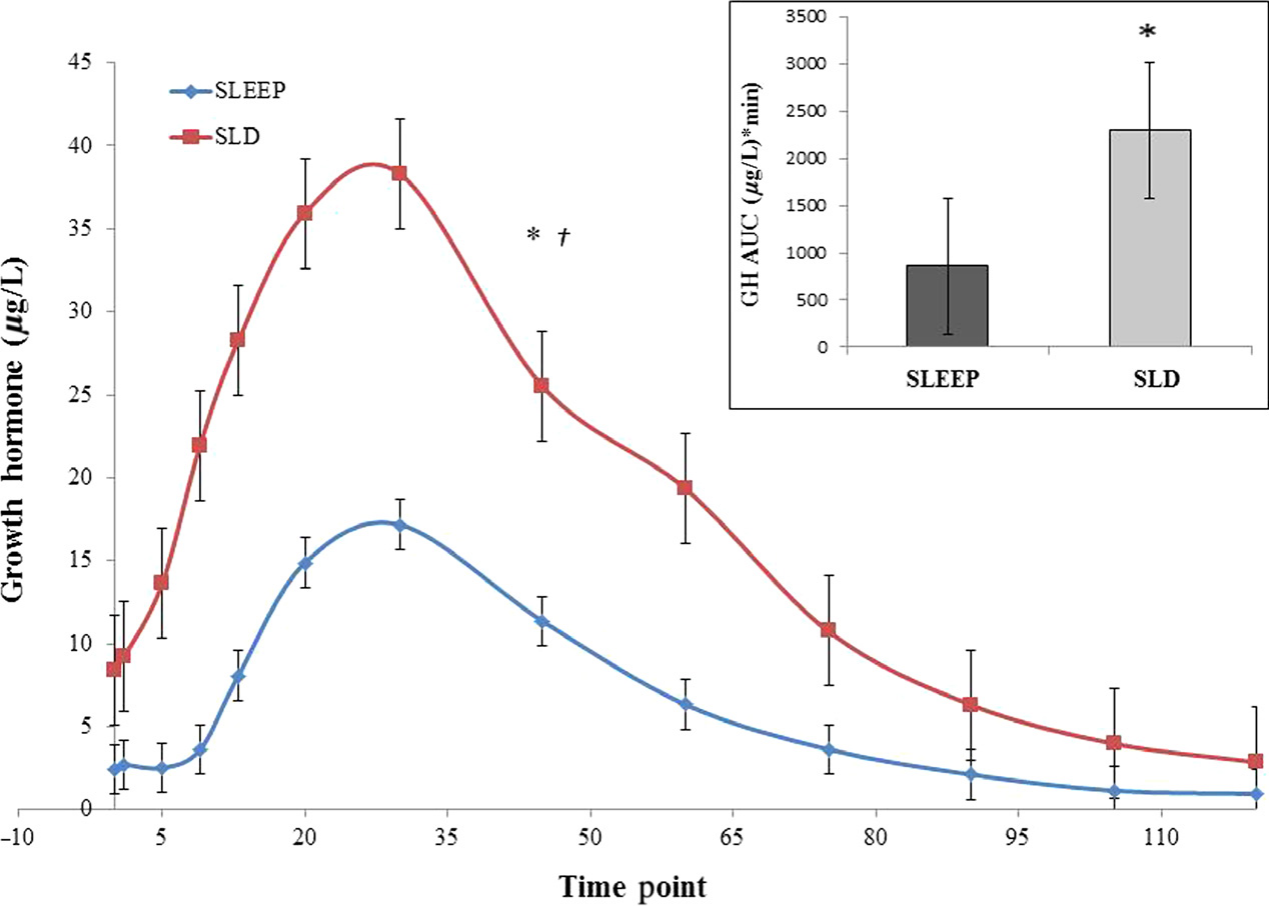
Mean GH concentrations at each time point between SLEEP and SLD sessions during exercise and recovery. *Results indicated a significant interaction effect between time point and session (*P* < 0.05), and main effects for time point (*P* < 0.01) and session (*P* < 0.05). †Wilcoxon signed‐rank tests indicated that exercise‐induced GH concentrations were significantly lower at each timepoint during the SLEEP session from the onset of exercise (time point 0) through the remainder of the 120‐min profile. Total GH AUC was significantly greater during the SLD versus SLEEP session (*P* < 0.01).

GH AUC (exercise + recovery), peak GH concentration and ΔGH were significantly lower during the SLEEP session (*P *<**0.01) whereas resting GH release and time to reach peak GH concentration did not differ between sessions (Table 3). Results of the repeated measures ANOVA indicated a significant interaction effect between time point and session, *F* (1.957, 35.221) = 4.434, *P *<**0.05, η^2 ^= 0.198. There were also significant main effects for time point, *F* (1.957, 35.221) = 25.450, *P *<**0.01, η^2 ^= 0.586, and session, *F* (1, 18) = 7.858, *P *<**0.05, η^2 ^= 0.304. Figure 3 shows the mean GH concentrations during exercise + recovery, starting at exercise onset (0700 h, *i.e., time point 0*), that included four successive 30 s maximal sprints with 4 min of active recovery on a cycle ergometer. Wilcoxon signed‐rank tests were used to further examine which average GH concentrations (by time point) differed between sessions. Results indicated that exercise‐induced GH concentrations were significantly lower during the SLEEP session compared to the SLD session from the onset of exercise (*time point 0*) through the remainder of the 120‐min profile.

**Table 3.** Growth hormone concentrations (M ± SEM).

Variable	SLEEP	SLD	*P*	Adjusted *P*†
Resting GH (*μ*g/L)	0.57 ± 0.13	1.35 ± 0.55	0.181	0.575
Peak GH (*μ*g/L)	17.8 ± 3.7	39.6 ± 7.1	0.002*	
Time to Peak GH (min)	29.5 ± 2.2	27.0 ± 1.5	0.299	0.257
ΔGH (*μ*g/L)	17.2 ± 3.7	38.2 ± 7.3	0.003*	
GH AUC (*μ*g/L/min)^1^	825.0 ± 199.8	2212.8 ± 441.9	0.001*	

^1^ GH AUC during exercise and recovery only.

* *P *< 0.01.

† When data was non‐normally distributed, *P* values were adjusted for skewedness (>1.0) using the nonparametric Wilcoxon sign‐ranked test.

Individual GH AUC and peak GH concentrations increased significantly in all subjects even though there was a large degree of interindividual variation during both sessions. However, subjects stayed within the same approximate rank response order during both sessions and this was confirmed by the statistically significant test‐retest correlations between sessions for both GH AUC (*r *=**0.888, *P *<**0.01) and peak GH concentration (*r *= 0.845, *P* < 0.05).

### Free IGF‐1

**Figure 4.**
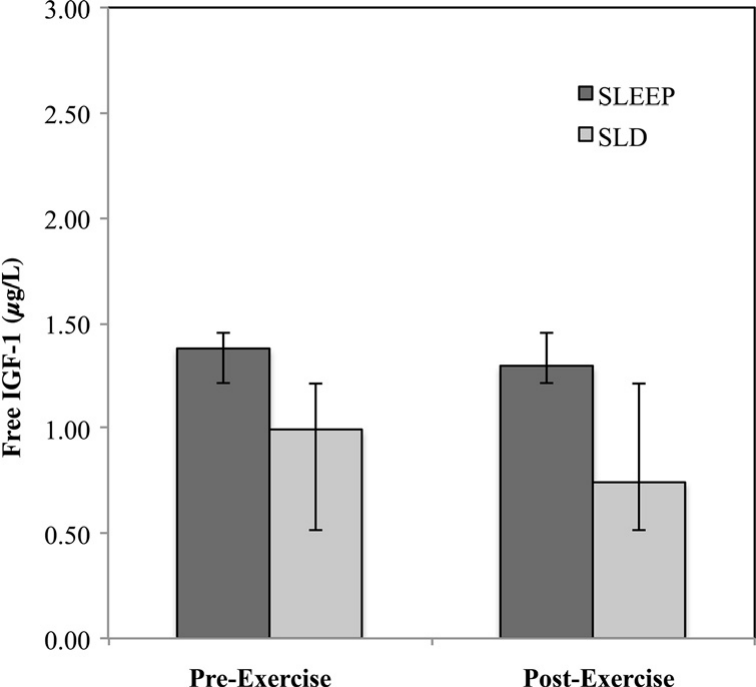
Free IGF‐1 response pre‐ and 90 min postexercise between sessions.

A repeated measures ANOVA indicated that free IGF‐1 was similar following exercise, *F* (1, 12) = 0.945, *P *=**0.350, η^2 ^= 0.073, and was also similar between sessions, *F* (1, 12) = 0.871, *P *=**0.429, η^2 ^= 0.053. Furthermore, there was no interaction effect of exercise and session, *F* (1, 18) = 0.228, *P *=**0.642, η^2 ^= 0.019 (Fig. 4).

### Lactate

Exercise significantly increased blood lactate concentrations over resting values during both SLD (M ± SEM) (1.49 ± 0.14 vs. 15.79 ± 1.40 mmol/L, *P *<**0.01) and SLEEP sessions (1.54 ± 0.19 vs. 17.15 ± 1.54 mmol/L, *P *<**0.01). However, no differences in blood lactate concentrations existed between SLD and SLEEP sessions at rest (1.49 ± 0.14 vs. 1.54 ± 0.19 mmol/L, *P *=**0.811) and immediately following exercise (15.79 ± 1.40 vs. 17.15 ± 1.54 mmol/L, *P *=**0.467). The univariate analysis of variance revealed no interaction effect of exercise and session on blood lactate concentrations (*P *=**0.533).

## Discussion

The primary findings from the current study can be summarized as follows 1) early morning resting GH concentration was unaffected by sleep deprivation, and 2) exercise‐induced GH AUC (168%), peak GH concentration (123%) and Δ GH (122%) were significantly greater following a night of sleep deprivation.

The present study supports previous research that suggests short‐term, high‐intensity exercise augments the GH response following a night of adequate sleep (Nevill et al. 1996; Kanaley et al. 1997; Stokes et al. 2002a,b, 2003, 2004, 2005; Stokes 2003) and partial sleep deprivation (Abedelmalek et al. 2013). It has been proposed that intense physical activity combined with caloric and sleep restriction can amplify GH secretion (Nindl et al. 2003). However, few studies have attempted to examine the singular effects of acute sleep deprivation on exercise‐induced GH release. Abedelmalek et al. (2013) reported that growth hormone was increased following partial sleep deprivation using repeated brief sprint exercises similar to the current study. In Abedelmalek et al. (2013), thirty healthy, college‐aged athletes exercised at 0800 h after only being allowed to sleep from 2230 to 0300 h compared to our complete lack of overnight sleep during the SLD session. During each of their exercise sessions, subjects completed 4 x 250‐m runs on a treadmill at a constant intensity of 80% of an individualized maximal speed with 3‐min recovery intervals. However, Abedelmalek et al. (2013) failed to report significant elevations in GH immediately postexercise due to lack of postexercise followup time points and the fact that the exercise‐induced GH response was much less than the current study. These differences are likely due to the partial and not complete (24‐h) sleep deprivation utilized by Abedelmalek et al. (2013) and its subsequent effects on nocturnal GH release and feedback mechanisms. For example, Mougin et al. (2001) reported that partial sleep deprivation did not affect exercise‐induced GH release. However, in this study, subjects were awoken as soon as they were about to enter rapid‐eye movement (REM) sleep unlike Abedelmalek et al. (2013) that allowed subjects to sleep for 4 consecutive hours. They were kept awake by pursuing sedentary activities in bed for 3 h before being allowed back to sleep until 0700 h and remained sedentary until their exercise session at 0200 h. Within a normal episode of sleep, the first four stages constitute NREM) sleep and are made up of several cycling stages of consciousness and slow‐wave brain activity. Typically, most GH release occurs during this NREM phase constituting slow‐wave sleep (SWS) that occurs within these first 4 h (Takahashi et al. 1968; Parker et al. 1969; Sassin et al. 1969; Karacan et al. 1971; Van Cauter et al. 1992a,b) with little GH secreted during subsequent REM sleep (Holl et al. 1991). Thus, subjects in the Mougin et al. (2001) and Abedelmalek et al. (2013) studies were not sleep deprived during the SWS phase when most nocturnal GH release occurs and this is likely the reason why exercise‐induced GH release was similar in both admissions. This is in agreement with a much earlier study by Karacan et al. (1971), who reported lower GH concentrations during sleep deprivation in which subjects were awakened prior to entering SWS. Based on these previous observations examining exercise‐induced GH release during partial sleep deprivation, we believe the current investigation is the first to report the response of growth hormone to short‐term, high‐intensity exercise following acute (24‐h) sleep deprivation.

Total GH AUC and peak GH concentration during the SLEEP condition were similar to previously reported research (Stokes et al. 2002a,b, 2003, 2004, 2005, 2010; Stokes 2003) but during the SLD session they were increased 2.7‐fold and 2.2‐fold, respectively. Our subjects were not sprint‐trained and had exercise‐induced peak GH concentrations during SLEEP that were similar to endurance trained males during a 30‐sec treadmill sprint (17.8 vs. 15.9 *μ*g/L, respectively). However, when the same subjects were sleep deprived, their exercise‐induced peak GH concentration were only slightly lower than the maximal peak GH values recorded in sprint‐trained males (39.6 vs. 44.0 *μ*g/L, respectively) (Nevill et al. 1996). This suggests that the capacity for GH synthesis and storage in sprint‐trained individuals may be augmented in a manner that is similar to what is observed during acute SLD and when a sprint exercise stimulus is provided, the magnitude of the GH response is similar regardless of training status. However, the mechanisms regulating GH synthesis and storage in these two scenarios (sprint‐trained vs. sleep deprived) are likely to be different and are yet to be fully elucidated.

Despite these large differences between the SLEEP and SLD conditions, there was still a large interindividual variation in GH concentration and peak GH. Test–retest (Pearson) correlations of GH AUC and peak GH concentration between sessions in the current study were similar to those that were reported by Stokes et al. (2003) (ranging from *r *=**0.89 to 0.97, *P *<**0.05). Stokes et al. (2003) reported significant test–retest correlations when subjects performed one 30‐s all‐out sprint followed by 60 min of recovery on two separate occasions, separated by 7 days. However, our test–retest correlations were similar across two separate conditions (SLEEP vs. SLD). This likely represents the rank response order within subjects (i.e., those that a large GH response during the SLEEP session also had a large, yet amplified GH response during the SLD session). Therefore, the slightly lower test–retest correlations in our study depict the augmented GH response related to sleep deprivation.

In the current study, peak power was greater in the first sprint of the SLEEP versus SLD session; although the overall mean across all four sprints was similar between sessions. However, it is unlikely that the minor difference in exercise performance between the sessions allowed a signficant contribution of feedback to the neuroendocrine system (i.e., somatotropic axis) from the localized muscle activation. This is strengthened by the fact that the time to reach the peak GH concentration did not differ between sessions and was similar to previous reports using high‐intensity sprint exercise (Stokes et al. 2002b, 2003, 2005). This suggests that the neuroendocrine response related to the onset of exercise appears unaffected by sleep deprivation. Likely the large, acute stress of high‐intensity exercise on the neuroendocrine system was sufficient to override any lingering effects that sleep deprivation had on the system's regulation.

During exercise recovery, GH typically returns to baseline within 2 h regardless of aerobic or high‐intensity exercise (Wideman et al. 1999, 2000a,b; Pritzlaff‐Roy et al. 2002; Stokes et al. 2002a; Weltman et al. 2006). Data from exercise recovery during the current study suggests that the neuroendocrine systems related to recovery were also unaltered during sleep deprivation. In the current study, GH concentrations were significantly elevated compared to rest for 60‐min postexercise in both sessions (Fig. 3) and this is similar to the previously mentioned study using short‐term, high‐intensity exercise (Abedelmalek et al. 2013). Since we cannot conclude that short‐term, high intensity exercise during sleep deprivation results in greater exercise‐induced GH recovery time beyond the expected postexercise period (i.e., 60‐min), we can suggest that (1) the half‐life of GH is unaffected by sleep deprivation and (2) the increase in GH AUC is a result of a greater total GH output over the same time frame.

Few studies have evaluated the effects of brief sprint interval exercise on IGF‐1; however, Meckel et al. (2009) reported that GH concentrations increased with no subsequent change in IGF‐1 up to 1 h postexercise after subjects completed 4 successive sprints similar to the current study. In addition, IGF‐1 has been shown to be unaffected during 3 nights of subsequent delta‐wave sleep interruption (Older et al. 1998), however partial sleep deprivation has been shown to decrease total IGF‐1 concentrations only when combined with severe energy deficit caused by caloric deprivation and arduous physical activity (Alemany et al. 2008). In the current study, we controlled dietary intake so that it was standardized within subjects and between sessions with a focus on avoiding caloric restriction, which may explain why free IGF‐1 was unaffected in the current study. While IGF‐1 is known to exert a negative long‐loop, multilevel feedback effect on GH release (Le Roith et al. 2001), free IGF‐1 values were not influenced by exercise or sleep deprivation in the current study and therefore, this long‐loop, multilevel feedback was likely not a primary contributor to the enchanced GH response observed during SLD. More research needs to be conducted on the primary effects of exercise during sleep deprivation on GH‐mediated IGF‐1 without the confounding effects of caloric restriction and negative energy balance.

Our results demonstrate that short‐term, high‐intensity exercise is a very strenuous stimulus capable of enhancing GH release with or without sleep deprivation. While nocturnal GH release was not assessed in the current study, we assume that nocturnal GH release was minimal during sleep deprivation. Results support our hypothesis that short‐term high‐intensity exercise appears to override the sleep deprivation‐induced mechanisms that blunt GH release, resulting in a maximal GH response when exercise follows sleep deprivation. Metabolic effects of GH may potentially augment glucose availability for priority tissues, such as neuronal activity in the brain, during stressful states such as sleep deprivation, caloric restriction, and negative energy balance. Additionally, the ability of sleep deprivation to alter the exercise‐induced GH pulsatility profile could have substantial downstream GH‐mediated biological effects during the days following these types of events. When the innate sleep schedule is disrupted, 24‐h GH pulsatility is more sporadic and frequent “bursts” of GH released throughout a 24‐h period transpires to make up for the significantly lower and unassociated GH pulse that occurs at sleep onset (Brandenberger and Wiebel 2004). GH regulates fat metabolism, which influences body composition and insulin sensitivity. It has been demonstrated that lipolysis is upregulated in the presence of larger intermittent but not smaller continuous GH infusion (Laursen et al. 2001). If in fact exercise during sleep deprivation does normalize GH pulsatility patterns, then humans with disrupted sleep schedules may benefit metabolically by exercising following sleep deprivation, creating a more uniform intermittent pulse of GH release versus a more sporadic 24‐h GH release pattern. These substantial downstream GH‐mediated biological effects could lead to improvement in body composition, glucose tolerance or even muscle growth and repair for sleep‐deprived individuals (i.e., students, athletes, doctors, nurses, parents of newborns).

In conclusion, we have demonstrated that exercise can override the attenuation of the GH release during sleep deprivation to elicit a maximal GH response greater than exercise following a night of adequate sleep. Future research should examine the interaction between exercise and GH release mediators during sleep deprivation as well as the capability of exercise to normalize 24‐h GH pulsatility (and subsequent metabolic consequences) when normal sleep schedules are disrupted. Additionally, little is known about the effects of the exercise‐induced GH response during sleep deprivation and whether or not it “hyper‐activates” an auto‐negative feedback mechanism to suppress its own release throughout the day, so that total 24‐h GH AUC is similar to that of a 24‐h sleep‐deprived session without exercise.

## Conflict of interest

Ritsche K, Nindl BC and Wideman, L have nothing to declare.
